# Comparison of Fluidic and Non-Fluidic Surface Plasmon Resonance Biosensor Variants for Angular and Intensity Modulation Measurements

**DOI:** 10.3390/s23249899

**Published:** 2023-12-18

**Authors:** Piotr Mrozek, Lukasz Oldak, Ewa Gorodkiewicz

**Affiliations:** 1 Faculty of Mechanical Engineering, Bialystok University of Technology, Wiejska 45C, 15-351 Bialystok, Poland; 2 Bioanalysis Laboratory, Faculty of Chemistry, University of Bialystok, Ciolkowskiego 1K, 15-245 Bialystok, Poland; l.oldak@uwb.edu.pl (L.O.); ewka@uwb.edu.pl (E.G.)

**Keywords:** Surface Plasmon Resonance (SPR), SPR biosensor, non-fluidic and fluidic biosensor, angular modulation, intensity modulation

## Abstract

Fluidic and non-fluidic surface plasmon resonance measurements were realized for the same type of sensory layer and using the same mouse IgG antibody and anti-mouse IgG antibody biomolecular system. A comparison of the thicknesses of the anti-mouse IgG antibody layers bound to the ligand at increasing analyte concentrations ranging from 0.0 μg mL^−1^ to 5.0 μg mL^−1^ in the non-fluidic and the fluidic variant showed that the thickness of the bound anti-mouse antibody layers in the fluidic variant was approximately 1.5–3 times larger than in the non-fluidic variant. The greater thicknesses of the deposited layers were also reflected in the larger increment of the resonant angle in the fluidic variant compared to the non-fluidic variant in the considered range of analyte concentrations. The choice between fluidic and non-fluidic surface plasmon resonance biosensors may be justified by the availability of analyte volume and the intended modulation technique. When working with limited analyte, non-fluidic biosensors with intensity modulation are more advantageous. For larger analyte quantities, fluidic biosensors with angular modulation are recommended, primarily due to their slightly higher sensitivity in this measurement mode.

## 1. Introduction

Surface Plasmon Resonance (SPR) serves as a reliable platform for clinical analysis, offering minimal sample preparation and quantitative responses in label-free detecting of biomolecular interactions [1,2,3,4,5,6]. It competes with conventional antigen-antibody tests and optical detection methods. This technology enables the real-time detection and monitoring of biomolecular events. A significant advantage of SPR is its capability to analyze diverse biological samples without interfering with the analyte, allowing for biosensor reuse in subsequent evaluations. SPR’s flexibility in assay design and its requirement for low sample quantities make it highly valuable, with excellent sensitivity typically in the picomolar to nanomolar range for analyte detection [7,8,9].

The Kretschmann configuration is a frequently employed setup in SPR systems, where a biosensor is created either on a prism coated with nanoscale metal, usually gold, or more commonly on a glass slide coated with nanoscale metal and affixed to the prism [1,2,3,4,5,6,7,8,9]. Due to differences in measurement environments, the SPR method has two variations: fluidic and non-fluidic [10,11,12,13,14,15]. The non-fluidic technique can be distinguished from the traditional fluidic SPR method in two ways: (1) the biosensor is formed ex situ, while in classic SPR it is formed in situ during measurement; (2) in non-fluidic SPR, the SPR measurement occurs after the removal of processing liquids, whereas in classic SPR, it takes place while processing liquids are present. Fluidic SPR is the prevailing technique in analytical applications, where the sample flows through a measuring cell, and the SPR signal is continuously measured. A biosensor is created in real-time during measurement, and a cleaning solution is used for biosensor regeneration. Usually, two parallel channels are employed, and the analytical signal is determined based on the highest sensor-gram value. Non-fluidic measurements are typically conducted in a stationary setup with an array of separate measuring points to enhance result precision. Multi-sample measurements and chip regeneration are usually carried out. Unlike fluidic measurements, non-fluidic SPR measurement involves gentle drying of the biosensor.

Presently, the fluidic SPR method is the most widely employed SPR technique in the field of medical diagnostics, and its commercial growth is on the rise. Several companies, including Biacore AB, Jandratek GmbH, IBIS, BuoTul AG, and Affinity Sensors, are now producing various devices based on SPR technology [5]. SPR Imaging (SPRi) involves converting the SPR signal into an image using a CCD camera. Both classical SPR and SPRi have their place in instrumental solutions, with the imaging version often associated with non-fluidic measurements.

There is currently no published information directly comparing fluidic and non-fluidic SPR measurement methods [10]. This is primarily due to the fact that the vast majority of commercially available measurement devices, among which fluidic measurement instruments predominate, are designed to operate within a relatively narrow measurement range adapted to the chosen environment. The transition from a liquid environment, in which typical fluidic measurements are carried out, to an ambient air environment, in which measurements are carried out using a non-fluidic sensor after it has been dried, involves a significant change in the range of SPR resonance angle values. Typical devices are not designed to perform measurements with such a varying range of resonance angle values [5].

In this work, the comparison of fluidic and non-fluidic SPR measurement methods realized for the same type of sensory layer and biomolecular system is addressed for the first time. For this purpose, an SPR measurement apparatus designed to realize measurements over a wide range of resonance angles was used. The purpose of this research was to quantitatively compare the results obtained by the two methods and draw conclusions in terms of the similarities and differences that characterize the two methods.

## 2. Materials and Methods

### 2.1. SPR Apparatus

SPR experiments were conducted with a stationary apparatus developed jointly by the University of Bialystok and AC S.A (Poland). The key components of the SPR setup included a diode laser emitting light at a 635 nm wavelength, a fiber optic collimator, a linear polarizer, a glass prism and a chip configured in the Kretschmann configuration, and a CCD camera serving as a detector. Two polarizations were employed: *p* polarization for primary measurements and *s* polarization for background measurements, which were subsequently subtracted. The results were analyzed and processed using ImageJ software (NIH, version 1.32). The measurement range of the apparatus covers resonance angles (θ) ranging from 10 to 85 degrees. The apparatus design allows for the placement of both a multi-field chip for non-fluidic measurements and a chip for fluidic measurements on the prism in the Kretschmann configuration. The non-fluidic chip has nine circular measurement fields of 3 mm in diameter, arranged on a square base. The fluidic chip is equipped with two flow channels, 3 mm wide and 12 mm long, one for measurements and the other for reference purposes. For the non-fluidic analysis in one measurement field, only 3 μL of analyte is needed. Flow measurements are conducted with reagent flow rate set usually at 10 μL min^−1^.

### 2.2. Chip Manufacturing

A detailed description of the thin film deposition process to create the SPR chip was presented in [16]. In brief, glass substrates with dimensions 20 × 20 × 1 mm with a refractive index of n = 1.51 were cut from microscope slides (Thermo Scientific, Waltham, MA, USA). The glass plates were polished using an aqueous cerium oxide suspension. Substrate surfaces were cleaned using detergent, acetone, and isopropyl alcohol. After each use of a cleaning agent, the glass slides underwent rinsing and ultrasonic washing in deionized water. Thin metallic films were then deposited onto the glass surface via physical vapor deposition within an NA501 vacuum system at a vacuum range of 8 × 10^−6^–1 × 10^−5^ hPa at room temperature. Resistive evaporation sources in the form of molybdenum boats were employed. The glass plates were positioned on a rotating substrate holder. In the initial step, a 0.1 nm adhesive chromium (Cr) layer (99.9%) was deposited at an approximate rate of 0.02 nm s^−1^. Subsequently, approximately 44.8 nm of silver (Ag) (99.99%) was deposited at a rate of 0.08 nm s^−1^, followed by approximately 3.3 nm of gold (Au) (99.99%) at a rate of 0.01 nm s^−1^. The thickness of each layer and deposition rate were continuously monitored using a quartz crystal microbalance. Based on the multi-layer coating deposited on glass, biosensors were developed for both non-fluidic and fluidic measurements.

### 2.3. Non-Fluidic SPR Biosensor Manufacturing

**Figure 1:**
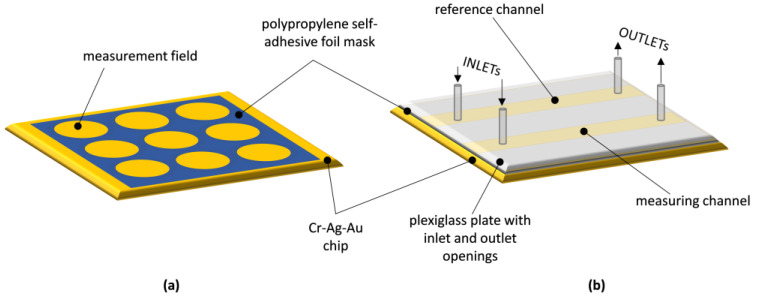
Schematic diagram of: (**a**) non-fluidic SPR biosensor; (**b**) fluidic SPR biosensor.

To create the non-fluidic biosensor, masks were used to determine the arrangement of measurement fields on its surface. These masks were crafted from a thin, single-sided polypropylene self-adhesive foil with a thickness of 20 μm, utilizing a punching method detailed in [13]. In brief, the number and spacing of the holes punched in the mask were controlled by a computer program that operated a numerically controlled device specially designed and manufactured for this purpose. The automated hole-punching process ensured precise hole spacing and reproducible mask production. The hydrophobic properties of the mask material played a crucial role in maintaining the separation of individual drops of analyte solution applied to the adjacent measurement fields of the mask, which were deposited on the sensor’s surface during its operation. A schematic of a non-fluidic SPR biosensor is shown in Figure 1a.

### 2.4. Fluidic SPR Biosensor Manufacturing

In order to make a fluidic biosensor, two longitudinal, parallel measurement fields for flow-based measurements were established on the surface of the metallic layer of the chip. This was achieved by using a mask made from double-sided polypropylene self-adhesive foil with a thickness of 30 μm (Figure 1b). The mask had two longitudinal openings running in parallel, and it was affixed to the metallic surface. A transparent plexiglass plate with inlet and outlet openings, aligning with the ends of the longitudinal openings in the foil mask, was positioned on the top surface of the mask. Applying pressure to the plexiglass plate over the mask resulted in an adhesive bond, forming a layered structure that allowed simultaneous reagent flow in two parallel channels created within the foil mask. These channels were defined by the upper surface of the plexiglass plate and the metallic layer of the chip, respectively. The spacing between the longitudinal channels of the flow-based sensor matched the dimensional distance between the circular measurement fields of the non-fluidic sensor. Reagent flow for fluidic measurements was accomplished using a dual-channel infusion pump with adjustable flow rates through small-diameter silicone tubes.

### 2.5. Reagents

The following reagents were used in the experimental part: normal mouse IgG antibodies FITC conjugate (0.06 mg mL^−1^) (SIGMA ALDRICH, Munich, Germany), anti-mouse IgG antibodies FITC produced in rabbit, IgG fraction of antiserum (10 mg mL^−1^) (SIGMA ALDRICH, Munich, Germany), N-hydroxy-succinimide (NHS) (SIGMA ALDRICH, Munich, Germany), N-Ethyl-N′-(3-dimethylaminopropyl) carbodiimide (EDC) (SIGMA ALDRICH, Munich, Germany), 11-Mercaptoundecanoic acid (MUA) acting as a linker (SIGMA ALDRICH, Munich, Germany), phosphate-buffered saline (PBS) pH = 7.4 (BIOMED, Lublin, Poland), acetate buffer pH = 3.50 (BIOMED, Lublin, Poland), carbonate buffer pH = 8.50–9.86 (BIOMED, Lublin, Poland). The solvents used to prepare solutions were absolute ethanol 99.8% (POCh, Gliwice, Poland) and MilliQ water (Simplicity**^®^** Millipore, Burlington, MA, USA).

Measurements were performed with one type of glass chip coated with thin film metal layers: with a 0.1 nm of chromium, 44.8 nm layer of silver and a 3.3 nm layer of gold (Bialystok University of Technology).

### 2.6. Procedure for Non-Fluidic and Fluidic Biosensor Preparation

The process of preparing the biosensors involved two main stages: the creation of a monolayer of MUA and the immobilization of mouse IgG antibodies.

In the initial step, glass chips coated with a metallic thin film underwent a thorough cleaning procedure, which included five washes with MilliQ water and absolute ethyl alcohol, followed by drying with an inert gas (argon). Subsequently, the cleaned and dried glass chips were submerged in a 2 mM alcohol solution of MUA for 12 h inside a glass container at room temperature. After this period, the chips were subjected to 10 rinse cycles, alternating between MilliQ water and absolute ethanol. Any remaining solvent was eliminated using a stream of argon.

In the second step, for the non-fluidic biosensor, following the application of a polymer mask onto the chip’s surface, sensory areas were activated by using a mixture of NHS (250 mM) and EDC (250 mM) in the presence of a carbonate buffer (pH = 8.5). After 1 min of mixing, this mixture was dispensed onto the active regions of the chip in the form of small droplets (3.0 μL). For the fluidic biosensor, activation was carried out by infusing the NHS-EDC mixture into both channels of the chip using a dual-channel infusion pump. After 15 min, the NHS-EDC mixture was rinsed away with PBS buffer, and subsequently, the mouse IgG antibody solution (0.06 mg mL^−1^) was applied to the measurement fields in a manner similar to the previous step for both types of sensors, respectively. The solution was then allowed to bond for 1 h. After this duration, the solution was removed by rinsing with PBS buffer, and the sensors were ready for measurements using anti-mouse IgG antibodies.

### 2.7. SPR Measurements Using Non-Fluidic and Fluidic Biosensors

SPR measurements were conducted using a specialized apparatus briefly outlined in SPR Apparatus section. The measurements involved determining the SPR angle, which corresponded to the point of maximum decrease in reflected light intensity R. The adsorption of anti-mouse IgG antibodies influenced the characteristics of the biosensor’s interface, causing a shift in the minimum angle, and the magnitude of this shift correlated with the concentration of the anti-mouse antibodies.

Measurements were conducted with analyte concentrations of 0.0 μg mL^−1^, 1.0 μg mL^−1^, 3.0 μg mL^−1^, and 5.0 μg mL^−1^ of anti-mouse IgG antibodies. Samples were prepared in PBS buffer. The resonance angle values were measured for both the fluidic and non-fluidic sensors at these analyte concentration levels.

For the non-fluidic biosensor, a 3.0 μL droplet of the analyte was placed in the measurement field for a duration of 15 min. Subsequently, the measurement field was rinsed by applying and removing 3.0 μL droplets of MilliQ water (repeated 10 times), and then the chip was dried using an argon stream. At least one measurement field for which no analyte was applied was used as a reference field.

In the case of the fluidic biosensor, the analyte flowed through the measurement channel for 15 min at a flow rate of 10 μL min^−1^. Then, the measurement field was rinsed with a stream of PBS buffer for 15 min at the same flow rate. The reference channel only had a flow of PBS buffer at a rate of 10 μL min^−1^.

### 2.8. SPR Curves Modeling

Winspall 3.02 software (RES-TEC Resonant Technologies GmbH) was employed for simulating SPR curves. This was utilized to derive the theoretical values of parameters characterizing optical layers by fitting resonance curves to the experimental data obtained through the Kretschmann configuration measuring system.

## 3. Results

**Figure 2:**
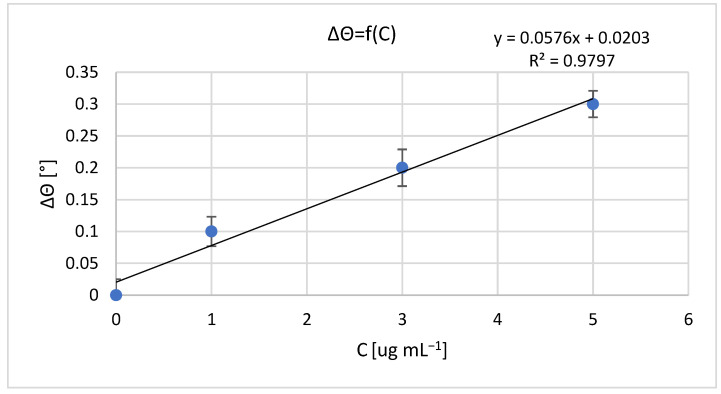
Calibration curve of mouse IgG antibody–anti-mouse IgG antibody biomolecular system for non-fluidic sensor; resonant angle for an analyte concentration of 0.0 μg mL^−1^: θ_0_n-f_ = 34.0°. Error bars are standard deviation (SD) calculated from 3 measurement repetitions.

**Figure 3:**
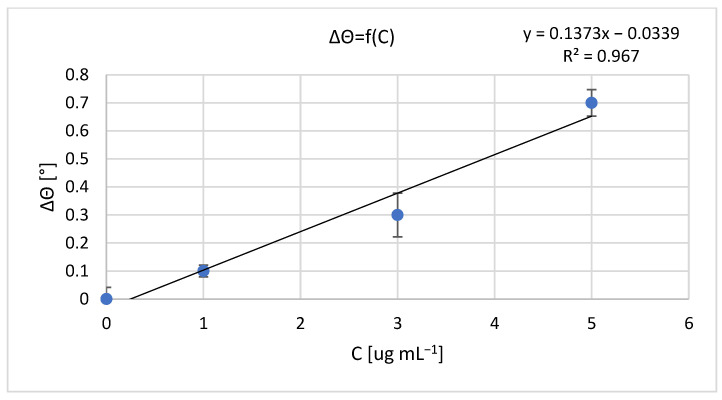
Calibration curve of mouse IgG antibody–anti-mouse IgG antibody biomolecular system for fluidic sensor; resonant angle for an analyte concentration of 0.0 μg mL^−1^: θ_0_f_ = 74.5°. Error bars are standard deviation (SD) calculated from 3 measurement repetitions.

The results of measurements obtained with a non-fluidic and fluidic sensor are shown in Figure 1 and Figure 2. For an analyte concentration of 0.0 μg mL^−1^, the resonant angle was equal to θ_0_n-f_ = 34.0° and θ_0_f_ = 74.5°, for the non-fluidic and fluidic sensor, respectively. Calibration curves are presented in Figure 2 and Figure 3, showing the change in the SPR angle as a function of analyte concentration for analyte concentration changes ranging from 0.0 μg mL^−1^ to 5.0 μg mL^−1^. The linear range covered the entire range of applied analyte concentrations, with regression coefficients R^2^ = 0.979 and R^2^ = 0.967 for non-fluidic and fluidic measurements, respectively. Limit of detection (LOD) and limit of quantification (LOQ) were calculated using the standard deviation (SD) based on the blank sample analysis using the following relationships: LOD = 3 × SD and LOQ = 3 × LOD. The calculated values are as follows: for the non-fluidic biosensor LOD = 0.075 µg mL^−1^, LOQ = 0.226 µg mL^−1^ and for the fluidic biosensor LOD = 0.125 µg mL^−1^, LOQ = 0.375 µg mL^−1^.

**Figure 4:**
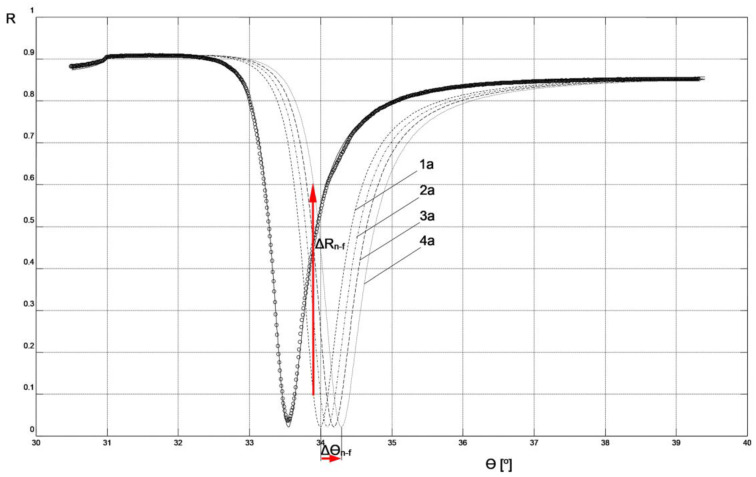
SPR model curves fitted to experimental results for non-fluidic sensor: circles—reflectance R measurement results for Cr-Ag-Au chip; solid line—curve of Cr-Ag-Au chip; 1a, 2a, 3a, 4a—curves corresponding to the parameters and measurement conditions included in Table 1.

In Figure 4, the results of reflectance R measurements corresponding to the SPR curve of the chip (only with Cr, Ag, and Au metallic layers) in an air environment are presented. These results were used to determine the modeling parameters of the SPR curve for this chip using WinSpall software. The parameter values used in the modeling are presented in Table 1, and the effect of fitting the model SPR curve to the experimental results is visible in Figure 4. The thicknesses of the metallic layers correspond to the results of measurements using the quartz crystal microbalance method conducted during the deposition of these layers in the PVD process.

**Figure 5:**
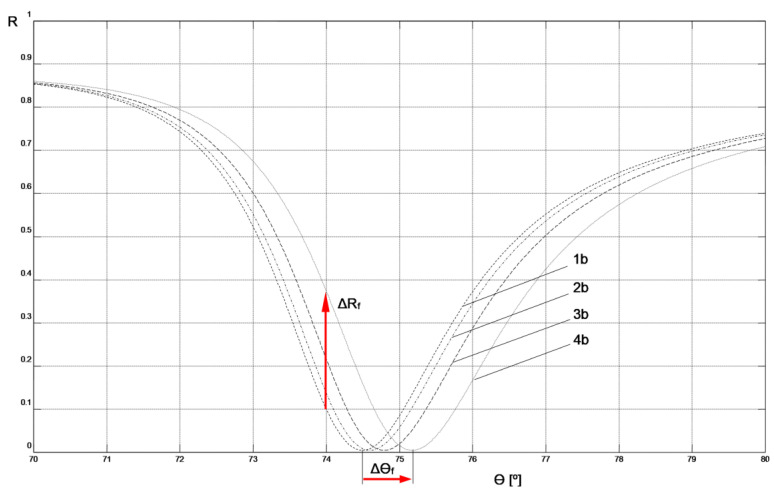
SPR model curves fitted to experimental results for fluidic sensor: 1b, 2b, 3b, 4b—curves corresponding to the parameters and measurement conditions included in Table 2.

The parameters of the Cr-Ag-Au chip layers obtained through modeling its SPR curve were used to model systems with layers deposited on the chip’s surface in the next step to create a biosensor (layers: MUA, mouse IgG antibody, anti-mouse IgG antibody, air, or PBS buffer). This was done in such a way that the resonant angle values of the modeled SPR curves of the system, as shown in Figure 4 and Figure 5, numerically matched the experimentally determined resonant angle values shown in Figure 2 and Figure 3.

The values of the real (ε′) and imaginary (ε″) part of the dielectric function for MUA [17], mouse IgG antibody, and anti-mouse IgG antibody [18], used in modeling the biosensor layers, were adopted in accordance with literature data regarding the values of the real (n) and imaginary (κ) part of the refractive index for these materials and the relationship (n + iκ)^2^ = ε′ + iε″. The ε′ value for the PBS buffer was calculated by assuming n = 1.34 in accordance with literature data [19]. Due to the identical preparation of MUA layers in both biosensor variants, the same layer parameter values were assumed for them. The thickness of this layer was assumed to be consistent with literature data [20]. Because of the methods used to immobilize the ligand on the biosensor surfaces under conditions of relatively high concentrations of mouse IgG antibodies and a long binding time with MUA in the preparation procedures of both types of biosensors, the same parameters for these layers were adopted in the modeling of both non-fluidic and fluidic biosensors. The thicknesses of the mouse IgG antibody layers were determined to achieve agreement in the resonant angle values of the modeled SPR curves without analyte with the experimental values for both types of sensors (SPR curves 1a and 1b in Figure 4 and Figure 5, respectively). This agreement was achieved with the same thickness value for the mouse IgG antibody layer for both biosensor variants, which is a strong argument in favor of the modeling assumptions made.

The parameter values for the layers deposited on the chip, used for modeling the SPR curves shown in Figure 4 and Figure 5, are presented in Table 2 and Table 3, respectively.

## 4. Discussion

By following the procedure described above, the result of the analyte interaction with the sensing layer of both types of biosensors could be modeled by adjusting only the thickness of the anti-mouse IgG antibody layer in such a way as to achieve agreement in the resonant angle values of the model SPR with the experimentally measured values (indicated in bold in Table 2 and Table 3). Simultaneously, the measurement environment was taken into account by adopting the parameters of air or PBS buffer for the simulation of non-fluidic or fluidic sensors, respectively. A comparison of the thickness of the anti-mouse IgG antibody layers bound to the ligand at increasing analyte concentrations ranging from 0.0 μg mL^−1^ to 5.0 μg mL^−1^ in the non-fluidic variant (corresponding to SPR curves 1a, 1b, 1c, and 1d in Figure 4 and Table 2) and the fluidic variant (SPR curves 2a, 2b, 2c, and 2d in Figure 5 and Table 3) shows that the thickness of the bound anti-mouse antibody layer in the fluidic variant is approximately 1.5–3 times larger than in the non-fluidic variant. The greater thickness of the deposited layer is also reflected in the larger increment of the resonant angle Δθ_f_ = 0.7° in the fluidic variant compared to Δθ_n-f_ = 0.3° for the non-fluidic variant in the considered range of analyte concentrations.

It appears that the cause of these quantitative differences between the two sensor variants can be attributed to the characteristic features of the analyte binding stage, which are different for both sensor types. The use of a single 3.0 μL analyte drop under static conditions for one measurement field of non-fluidic sensor results in less availability of the antibodies for binding to the ligand, compared to the more dynamic binding conditions of the fluidic sensor version and the larger working volume of the analyte flowing at a rate of 10 μL min^−1^ for 15 min in contact with the measurement spot in the sensor’s channel. In the case of a single drop, under established process conditions, the flow of antibodies towards the sensing layer is mainly diffusive. Under flow conditions, in addition to the diffusive character, there is a mass transport component associated with the flow rate. In these conditions, it appears to be dominant, despite the fact that, under steady-state flow, only the part of the analyte closest to the sensory surface has the ability to form bonds with it. So, an increased volume of flowing analyte contributes to enhancing the sensitivity of the SPR signal. At the same time, however, the occurrence of a flow of a certain intensity prevents the deposition of some analyte molecules in the active area of the sensor, negatively affecting its sensitivity. It seems that conducting a series of measurements at different flow rates would allow determining its optimal value.

Figure 4 and Figure 5 depict the values of Δθ_n-f_ = 0.3° and Δθ_f_ = 0.7°, representing changes in the resonant angle of the SPR curves for non-fluidic and fluidic biosensor variants, respectively, corresponding to analyte concentration changes Δc = 5 μg mL^−1^. These numerical values can be used to compare the sensitivities, S_ac_, of both biosensors used for measurements in the angular modulation variant [16]. In this variant, the average sensitivity of the sensor within the given range of analyte concentrations is calculated as S_ac_ = Δθ/Δc. Utilizing these numerical values, it is easy to observe that the sensitivity S_ac_f_ of the fluidic sensor is 2.3 times higher than the sensitivity S_ac_n-f_ of the non-fluidic sensor.

Figure 4 and Figure 5 also present the values of ΔR_n-f_ = 0.507 and ΔR_f_ = 0.269, representing changes in reflectance in the linear portions of the resonance curves for non-fluidic and fluidic biosensor variants, respectively, corresponding to analyte concentration changes Δc = 5 μg mL^−1^. These numerical values can be used to compare the sensitivities, S_ic_, of both biosensors used for measurements in the intensity modulation variant. In this variant, the average sensitivity of the sensor within the given range of analyte concentrations is calculated as S_ic_ = ΔR/Δc. By using these numerical values, it can be observed that the sensitivity S_ic_n-f_ of the non-fluidic sensor is 1.9 times higher than the sensitivity S_ic_f_ of the fluidic sensor. The comparison result suggests that, in the case of the non-fluidic sensor, it is more advantageous to perform measurements in the intensity modulation variant, while for the fluidic sensor it is more advantageous to perform measurements in the angular modulation variant.

Smaller increases Δθ in the resonance angle observed when increasing the analyte concentration, as measured in the case of a non-fluidic sensor, result from a thinner layer of anti-mouse antibody bound with the ligand compared to a similar layer for a fluidic sensor. Through simulations in Winspall software, it can be easily demonstrated that increasing the thickness of the anti-mouse antibody layer in a non-fluidic sensor to the value obtained for a fluidic sensor leads to an increase Δθ in the resonance angle by even slightly more than that for the fluidic sensor. Therefore, it is evident that all actions aimed at increasing the thickness of the bound analyte layer will have a positive impact on the sensor’s sensitivity, both in angular modulation and intensity modulation variants. It seems that optimizing process parameters. such as analyte binding time, temperature, pH, droplet volume (for a non-fluidic sensor), and flow rate (for a fluidic sensor). should result in achieving the maximum thickness of the bound layer.

Comparison of the SPR curves presented in Figure 4 and Figure 5 clearly indicates that the slope of the curve in the vicinity of the resonance angle is significantly greater in the case of the non-fluidic sensor. This characteristic of the SPR curve in its mathematical description arises from the real part ε′ of the dielectric function of the air environment adjacent to the layer of analyte bound to the surface of this type of sensor. This feature favors the use of the sensor in this variant for conducting measurements with intensity modulation, because relatively small increases Δθ in the resonance angle significantly affect the increase in reflectance ΔR at a fixed angle θ (Figure 4). At the same time, it can be stated that increasing the resonance angle increment Δθ of the non-fluidic sensor by increasing the thickness of the bound layer, for example, to values obtained in the fluidic variant, would significantly contribute to enhancing the sensitivity of measurements with intensity modulation. This is potentially achievable in practice by binding a thicker layer of analyte to the surface of a fluidic sensor, then removing the liquid from its surface, drying it, and conducting measurements with intensity modulation as described for the non-fluidic variant. Such a measurement approach has not been described in the literature so far. Currently available SPR devices are not technically prepared for such a procedure. Nevertheless, based on the results presented in this study, it can be concluded that such a measurement approach would exhibit the highest sensitivity using intensity modulation. Verification of this hypothesis will be the focus of future research after the necessary adaptation of the measurement system.

It appears that the characteristics of the mouse IgG antibody system are broad enough to ensure that the test results are not restricted in their relevance to other biomolecular systems. In the case of alternative analyte binding variants compared to the carboxyl group activated with EDC/NHS used in this study, such as binding using a thiol or aldehyde group, it seems that the described factors influencing the thickness of the bound analyte layer should play a similar role. Therefore, they should similarly affect sensitivity in both measurement variants.

## 5. Conclusions

The comparison of biosensor sensitivities in non-fluidic and fluidic variants, as well as in angular modulation and intensity modulation, indicates under which circumstances it would be more advantageous to use a specific variant. It seems that, in cases where only a small amount of analyte is available, sufficient only for preparing a small number of drops with small volumes, it would be more beneficial to use a non-fluidic biosensor with intensity modulation. For situations with a larger quantity of available analyte, it would be more advantageous to use a fluidic biosensor with angular modulation, also considering its slightly higher sensitivity in this sensor variant. Therefore, the non-fluidic version is almost exclusively applied for analytical purposes, but the fluidic version requires the signal enhancement for such a purpose. It also seems that the availability of measurement equipment dedicated to both non-fluidic and fluidic measurements, as well as the level of automation in the system and the workload involved in both sensor variants, may play a significant role in choosing one of these measurement methods. The fluidic variant is generally characterized by a lower degree of labor intensity in conducting measurements and, given the availability of a larger quantity of analyte, may favor the selection of this variant.

In the Discussion section, a measurement approach was proposed that would exhibit the highest sensitivity of non-fluidic sensor using intensity modulation. Future research will focus on verifying this hypothesis after the necessary adaptation of the measurement system.

## Figures and Tables

**Table 1:** Parameters used to model the SPR curve of a Cr-Ag-Au chip using WinSpall software; ε′ and ε″—real and imaginary part of the dielectric function of the layer, respectively.

Layer	Thickness (nm)	ε′	ε″
microscope slide	∞	2.29	0.00
chromium	0.10	−2.10	20.90
silver	44.75	−19.40	1.00
gold	3.25	−12.80	1.40

**Table 2:** The parameter values of the SPR layers applied to the Cr-Ag-Au chip used to model the SPR curves shown in Figure 4 (non-fluidic sensor). Anti-mouse IgG layer thicknesses adjusted to match the model SPR resonance angle values with experimentally measured values are shown in bold.

SPR Curve No.	Analyte Concentration (μg mL^−1^)	Resonance Angle (^o^)	Layer	Thickness (nm)	ε′	ε″
1a	0.0	34.0	MUA	1.2	2.196	0.00
			Mouse IgG	2.4	1.96	0.00
			Air	∞	1.00	0.00
2a	1.0	34.1	MUA	1.2	2.196	0.00
			Mouse IgG	2.4	1.96	0.00
			Anti-mouse IgG	**0.8**	1.96	0.00
			Air	∞	1.00	0.00
3a	3.0	34.2	MUA	1.2	2.196	0.00
			Mouse IgG	2.4	1.96	0.00
			Anti-mouse IgG	**1.5**	1.96	0.00
			Air	∞	1.00	0.00
4a	5.0	34.3	MUA	1.2	2.196	0.00
			Mouse IgG	2.4	1.96	0.00
			Anti-mouse IgG	**2.3**	1.96	0.00
			Air	∞	1.00	0.00

**Table 3:** The parameter values of the SPR layers applied to the Cr-Ag-Au chip used to model the SPR curves shown in Figure 5 (fluidic sensor). Anti-mouse IgG layer thicknesses adjusted to match the model SPR resonance angle values with experimentally measured values are shown in bold.

SPR Curve No.	Analyte Concentration (μg mL^−1^)	Resonance Angle (^o^)	Layer	Thickness (nm)	ε′	ε″
1b	0.0	74.5	MUA	1.2	2.196	0.00
			Mouse IgG	2.4	1.96	0.00
			PBS buffer	∞	1.80	0.00
2b	1.0	74.6	MUA	1.2	2.196	0.00
			Mouse IgG	2.4	1.96	0.00
			Anti-mouse IgG	**1.1**	1.96	0.00
			PBS buffer	∞	1.80	0.00
3b	3.0	74.8	MUA	1.2	2.196	0.00
			Mouse IgG	2.4	1.96	0.00
			Anti-mouse IgG	**3.2**	1.96	0.00
			PBS buffer	∞	1.80	0.00
4b	5.0	75.2	MUA	1.2	2.196	0.00
			Mouse IgG	2.4	1.96	0.00
			Anti-mouse IgG	**7.4**	1.96	0.00
			PBS buffer	∞	1.80	0.00

## Data Availability

Data are contained within the article.
